# Rising CNS Polypharmacy Among Older Adults in the United States

**DOI:** 10.1093/geroni/igaa057.2689

**Published:** 2020-12-16

**Authors:** Donovan Maust

**Affiliations:** University of Michigan, Ann Arbor, Michigan, United States

## Abstract

CNS-polypharmacy is defined by the AGS Beers Criteria as using 3 or more individual medications from the following classes: antidepressants, antipsychotics, benzodiazepines, other sedative/hypnotics, opioids, antiepileptics. Dr. Maust will review data suggesting that such prescribing has increased among older adults, along with the data suggesting there are associated harms. In addition, he will review recent evidence from the Department of Veterans Affairs, which suggests that older Veterans who use both Medicare and the VA system for medical care are at higher risk of potentially inappropriate CNS-active prescribing. Part of a symposium sponsored by the Aging, Alcohol and Addictions Interest Group.

